# Dual Roles of Adipose Tissue in Skeletal Muscle Regeneration: Pro‐Regenerative Versus Maladaptive

**DOI:** 10.1002/jcsm.70269

**Published:** 2026-04-17

**Authors:** Chongxuan Lu, Feng Lu, Junrong Cai

**Affiliations:** ^1^ Department of Plastic and Cosmetic Surgery Nanfang Hospital, Southern Medical University Guangzhou Guangdong People's Republic of China

**Keywords:** adipokines, adipose tissue, adipose‐derived stem cells, browning, fatty infiltration, fibro‐adipogenic progenitors, skeletal muscle repair

## Abstract

Skeletal muscle accounts for approximately 40% of total body mass and is essential for locomotion, metabolic regulation and systemic homeostasis. Adipose tissue is increasingly recognized as an active component of the muscle's regenerative microenvironment. During muscle repair, adipose tissue contributes to local metabolic support and paracrine signalling, but its effects are highly context dependent. Tightly regulated presence of adipose tissue accompanies effective regeneration, whereas excessive or dysregulated ectopic fat accumulation leads to persistent functional impairment. This review synthesizes recent experimental and translational studies investigating the cellular origins, regulatory mechanisms and functional consequences of adipose tissue during skeletal muscle repair. We propose three potential therapeutic directions to improve pathological muscle repair by modulation of the adipose‐related intramuscular regenerative microenvironment: (1) targeted modulation of fibro‐adipogenic progenitors (FAPs) to curb pathological adipogenesis and fibrosis while leveraging beneficial mediators such as adiponectin and leptin; (2) adipose‐derived stem cell transplantation and exosome‐based approaches to deliver multipotent cells and remodel the repair microenvironment; and (3) induction of adipose browning to enhance energy availability, immune tone and vascularization while recognizing its context‐dependent liabilities. Together, these strategies position adipose tissue as a plastic and environment‐sensitive regulator of muscle regeneration and provide a framework for precision interventions to improve structural and functional outcomes.

## 1 Introduction

Skeletal muscle, which constitutes ~40% of body mass, is a highly heterogeneous tissue that consists of multinucleated myofibers derived from satellite cells, as well as multiple non‐myogenic cell populations, including but not limited to fibro‐adipogenic progenitors (FAPs), endothelial cells, immune cells and adipocytes, and exhibits substantial plasticity and regenerative capacity [S1]. Beyond locomotion, it is a key regulator of systemic metabolism and overall health. Nevertheless, this tissue is vulnerable to diverse challenges—including aging, obesity, acute trauma such as mechanical damage (e.g., contusion or laceration), ischemia–reperfusion, toxin exposure, or intense or unaccustomed exercise, as well as maladaptive or chronic repair processes following acute injury, and chronic degenerative disorders such as muscular dystrophies and sarcopenia [1, 2], [S2].

Skeletal muscle repair is a highly coordinated process that restores tissue structure and function through overlapping phases of inflammation, regeneration and remodelling [S3]. Initially, a tightly controlled inflammatory response clears necrotic debris and conditions the regenerative niche. Regeneration is driven by resident satellite cells (MuSCs), which re‐enter the cell cycle, proliferate, differentiate into myoblasts and fuse to rebuild damaged myofibers. Concurrently, FAPs, a population of mesenchymal progenitors, expand transiently, providing essential paracrine signals and extracellular matrix support that facilitate MuSC proliferation, differentiation and myofiber formation [S4, S5]. However, although minor injuries typically heal effectively, severe trauma, aging and chronic disease often result in incomplete repair. In these contexts, dysregulated healing—especially persistent inflammation—can bias FAPs toward fibrogenic or adipogenic fates, leading to fibrosis and fatty infiltration which means the generation of excessive ectopic fat deposition within the muscle, impairing functional recovery and contributing to sustained weakness, restricted range of motion and an increased risk of reinjury [2, 3, 4, 5].

Mammalian adipocytes comprise white (WAT), brown (BAT) and beige subtypes. White adipocytes, defined by a single large triglyceride droplet, function as the principal reservoir for fatty acids and modulate systemic metabolism and inflammation through adipokines such as leptin and adiponectin [6, 7]. Brown adipocytes harbour multilocular lipid droplets and abundant mitochondria and express uncoupling protein‐1 (UCP1), which short‐circuits the proton motive force across the inner mitochondrial membrane to generate heat and increase energy expenditure [S6, S7]. Beige adipocytes, located within WAT depots, exhibit inducible thermogenesis and acquire UCP1‐dependent activity in response to cold exposure, exercise or β3‐adrenergic stimulation [8].

Adipose tissue is increasingly recognized as a context‐dependent regulator of skeletal muscle repair, particularly adipose depots located within and in close proximity to skeletal muscle, where they are anatomically positioned to exert direct effects on the regenerative process. During homeostatic regeneration, a limited presence of adipocytes within and adjacent to skeletal muscle—and their adipokines (e.g., leptin and adiponectin)—provides paracrine support for satellite‐cell activation, survival and differentiation, whereas adipose‐derived stem cells (ADSCs) can be recruited to injury sites to augment local progenitor pools and promote repair [8, 9, 10, 11]. In chronic muscle disorders such as sarcopenia, muscular dystrophies and obesity‐associated myopathy, persistent inflammation and metabolic dysfunction drive excessive adipose expansion within skeletal muscle, creating a hostile microenvironment that suppresses myogenesis and impairs muscle regeneration [12, 13, 14].

Targeted modulation of FAPs is crucial for preventing maladaptive skeletal muscle repair. Timely clearance of excess FAPs through controlled apoptosis, coupled with restriction of adipogenic conversion, limits fibrosis and ectopic fat deposition while preserving their pro‐regenerative support for MuSCs [4]. Elucidating the signalling pathways that govern FAP fate—including TNF, NOTCH, WNT and mechano–endocrine axes—may provide the theoretical basis for stage‐specific and personalized interventions [4, 15, 16, 17]. ADSCs support regeneration by secreting immunomodulatory and pro‐angiogenic factors, restraining adipogenesis and, in some contexts, directly contributing to myogenesis [18, 19]. However, their effects are context dependent, necessitating strategies that steer lineage commitment toward myogenic and vascular fates while minimizing ectopic fat accumulation under metabolic stress [20]. Finally, adipose browning, through UCP1‐mediated thermogenesis and enhanced mitochondrial activity, improves energy availability, insulin sensitivity and angiogenesis, thereby limiting fatty infiltration [21, 22, 23]. However, as browning may exacerbate atrophy in catabolic states, careful regulation will be essential to balance its therapeutic potential against pathological risk [24].

Building on these advances, this review synthesizes current understanding of the roles and mechanisms of adipose tissue within and adjacent to skeletal muscle in the context of muscle repair, with a focus on its white‐ and beige‐like states and their dynamic interconversion. We highlight the regulation of FAPs' fate, the context‐dependent actions of adipokines and the contribution of ADSCs as facilitators of regeneration, and we discuss the reparative potential of adipose browning in shaping the intramuscular regenerative niche through pharmacological, exercise and nutritional strategies. Finally, we propose a ‘threshold’ hypothesis for intramuscular adipose tissue dynamics, in which limited, tightly regulated adipose accumulation supports regeneration, whereas expansion beyond a pathological threshold disrupts the regenerative niche. Together, these integrated insights provide a conceptual framework and translational guidance for precision modulation of the adipose‐related regenerative microenvironment.

## 2 Cellular Basis of Adipose Tissue Formation

Small adipocyte deposits are detectable within healthy skeletal muscle [9, 10]. Perivascular precursors—including PPARγ^+^ mural cells—contribute substantially to white adipocyte formation, supporting a key role for the vascular niche [25]. Recent single‐cell transcriptomic and lipidomic analyses further suggest that certain myeloid‐derived populations (e.g., Pdgfra^−^/Cd68^+^) may exhibit adipogenic capacity, although this evidence remains preliminary [26]. Collectively, these findings support a model in which physiological adipose formation in muscle emerges from multiple precursor lineages, shaped by local microenvironmental cues and systemic metabolic regulation.

In contrast, ectopic fat accumulation in chronic muscle disorders is consistently linked to dysregulated stromal progenitor fate, with FAPs representing a principal and experimentally tractable cellular source [13]. FAPs are muscle‐resident, non‐myogenic stromal cells most reliably identified by PDGFRα expression, and one feature of their heterogeneity is the adipogenic plasticity across distinct adipocyte‐like states. Under permissive pro‐adipogenic conditions, hedgehog signalling, changes in NOTCH and WNT/β‐catenin signalling, together with PPARγ, have been frequently associated with enhanced FAP adipogenesis, facilitating differentiation into white adipocyte–like cells with adipogenic marker expression and lipid accumulation comparable to canonical adipocyte precursors [4, 27], [S8–S10]. Beyond white adipogenesis, FAPs can also adopt brown or beige‐like adipocyte fates under defined contexts. This thermogenic potential has been linked to Prdm16‐centred regulatory programmes, thereby favouring brown/beige adipogenic differentiation in response to cues including β‐adrenergic stimulation [S11, S12]. Although FAPs represent a principal cellular source of ectopic adipocytes, they are not exclusive; the cellular and molecular underpinnings of ectopic adipocyte accumulation are complex, diverse and context‐specific.

## 3 Physiological Muscle‐Associated Adipose Tissue Is Indispensable for Muscle Development, Homeostasis and Regeneration

Genetic or pharmacologic inhibition of adipogenesis impairs regeneration, evidenced by reduced myofiber formation and attenuated satellite‐cell proliferation [7, 9, 10]. This suggests that physiological muscle‐associated adipose tissue supports muscle repair. A key challenge is distinguishing physiological from pathological adiposity, defining molecular features and assessing the degree of adipose accumulation.

Adipose tissue influences muscle function and regeneration in part through paracrine signalling, with leptin being the most extensively studied adipokine. Its receptor is broadly expressed in skeletal muscle. Leptin promotes repair by enhancing PGC‐1α activity, reshaping microRNA networks and activating STAT3‐dependent transcriptional programmes. Additionally, leptin increases FNDC5/irisin activity via a nitric oxide–dependent pathway, stimulating myoblast differentiation and improving muscle performance [28, 29, 30, 31]. Adiponectin also supports muscle homeostasis and repair through AMPK–PGC‐1α–dependent myogenesis and T‐cadherin‐mediated regulation of exosome secretion, reducing inflammation and fibrosis [32, 33]. During early regeneration, TNF‐α facilitates immune‐cell recruitment, clearance of necrotic tissue and activation of satellite cells [4, 27, 34]. IL‐6 enhances fatty‐acid oxidation and stimulates satellite‐cell proliferation during acute exercise and early repair [35, 36]. Irisin, secreted by both muscle and adipose tissue, promotes regeneration and fibre growth by activating satellite cells and protein synthesis [37]. Additional adipose‐derived factors contribute as well: VEGF drives angiogenesis in injured muscle, improving perfusion and indirectly supporting satellite‐cell activity and myofiber formation [38, 39]; IGF‐1 directly promotes satellite‐cell activation and hypertrophy [40, 41]; chemerin regulates terminal myoblast differentiation via CMKLR1, with pathway loss impairing muscle development [42, 43]; and apelin, upregulated by insulin, modulates energy metabolism and exercise adaptation, with the apelin–APJ axis protecting against atrophy and supporting regeneration under stress [44, 45]. However, these adipokine‐mediated benefits are highly context‐dependent, with chronic or sustained elevations potentially impairing regeneration.

Apart from paracrine signalling, adipose tissue serves as a reservoir of cells required for muscle repair. ADSCs derived from adipose depots adjacent to skeletal muscle home to injured muscle via a platelet‐dependent mechanism, supporting satellite‐cell expansion and myofiber regeneration; blocking ADSC recruitment impairs repair [11]. Adipose depots located within and in close proximity to skeletal muscle exhibit beige‐like differentiation after injury, secrete pro‐regenerative factors and facilitate myofiber differentiation and recovery [8, 21, 46]. Thus, adipose tissue contributes both by secreting adipokines and by supplying cellular populations for muscle reconstruction.

## 4 Pathological Ectopic Fat Accumulation as a Driver of Disease Progression in Skeletal Muscle Disorders

Across sarcopenia, muscle‐wasting disorders and chronic muscle injury–repair imbalance, ectopic fat accumulation within skeletal muscle emerges as a feature that integrates metabolic dysfunction, chronic inflammation and impaired regenerative capacity. Clinical and imaging studies consistently demonstrate that a greater intermuscular ectopic fat burden correlates inversely with muscle mass, strength and physical performance, predicting frailty, mobility limitation and adverse outcomes [1, 5, 12, 13, 17].

At the tissue level, intermuscular ectopic fat accumulation directly constrains skeletal muscle repair through its physical presence within the regenerative niche. As a non‐contractile tissue, intermuscular adipose tissue occupies space normally available for regenerating myofibers, thereby limiting fibre expansion and the re‐establishment of organized contractile architecture [13]. In parallel, adipose infiltration induces structural remodelling of muscle architecture: Fat deposition within interstitial and perimysial compartments alters muscle‐fibre orientation, pennation angle and fascicle length while increasing the stiffness of surrounding connective tissue, collectively reducing mechanical efficiency and force transmission [S13, S14]. Moreover, adipose accumulation around intramuscular vessels disrupts microvascular organization and impairs oxygen and nutrient delivery, further constraining the metabolic support required for effective repair [S15]. Together, these spatial, mechanical and vascular constraints establish intermuscular ectopic fat as a direct structural barrier to muscle regeneration.

The pathological impact of ectopic fat accumulation extends beyond its physical presence in muscle, as dysregulated adipokines drive an endocrine shift that links chronic inflammation and metabolic dysfunction to impaired muscle regeneration. Resistin exemplifies this pathogenic transition: Although initially implicated in host defence and tissue repair, chronically elevated resistin in aging, obesity and wasting conditions correlates with reduced muscle mass, strength and physical performance, and tracks disease severity in disorders such as COPD [47, 48, 49]. Mechanistically, resistin suppresses GLUT4 translocation and insulin‐ and AMPK‐stimulated glucose uptake, inhibits myogenic differentiation via NF‐κB–dependent pathways and amplifies local inflammation through induction of IL‐1β, IL‐6 and MCP‐1, thereby sustaining a catabolic microenvironment [50, 51, 52, 53]. Leptin, persistently elevated in obesity and chronic inflammatory states, constrains myoblast differentiation through sustained JAK/STAT and MEK/ERK signalling, promotes low‐grade inflammation via IL‐6 induction and biases macrophages toward a pro‐inflammatory M1 phenotype, collectively delaying regenerative resolution [14, 31, 54, 55]. Adiponectin, although generally anti‐inflammatory, may exert maladaptive effects in specific contexts: Hyperadiponectinemia in older adults is associated with muscle weakness, reduced mass and functional decline, and sustained receptor activation can suppress protein synthesis in experimental models [56, 57, 58]. Excessive TNF‐α further impairs regeneration by inducing NF‐κB–dependent MyoD degradation, inhibiting satellite‐cell differentiation and promoting mitochondrial dysfunction, thereby favouring muscle atrophy over effective repair [59, 60]. Chemerin, elevated in obesity and inflammatory states, disrupts muscle metabolism and myogenesis by inducing insulin resistance, inhibiting myogenic differentiation, promoting adipogenic transdifferentiation and triggering mitochondrial stress with excessive autophagy [61, 62, 63]. IL‐6, beyond serving as an inflammatory mediator, acts as a central driver of muscle catabolism when chronically elevated, sustaining STAT3‐dependent catabolic programmes and compromising regenerative efficiency [S16]. FGF21, frequently increased in hypermetabolic states such as cachexia, functions as a systemic metabolic stress signal; although acutely adaptive, its sustained elevation may exacerbate muscle energy deficits and limit metabolic support for regeneration [S17].

At the muscle‐intrinsic level, myostatin and activin act as potent negative regulators that suppress protein synthesis and myogenic differentiation, whereas follistatin serves as their endogenous antagonist that normally buffers this inhibitory drive [S18, S19]. When myostatin/activin signalling is sustained and/or follistatin antagonism is insufficient, the net balance shifts toward growth restraint and impaired regeneration, thereby weakening the muscle's ability to maintain its structural and metabolic dominance and creating a permissive environment for ectopic fat occupancy in sarcopenia and cachexia.

Among the adverse consequences of white fat accumulation, a particularly important factor is the induction of skeletal‐muscle insulin resistance, which undermines muscle maintenance, function and repair [S20]. Ectopic fat accumulation within skeletal muscle is closely associated with impaired insulin signalling. This disruption reduces glucose uptake and utilization, constraining metabolic support for regeneration, and also limits satellite‐cell proliferation and differentiation, thereby impairing myofiber formation, hypertrophy and functional recovery [S21, S22].

In cachexia, the relationship between adipose tissue and skeletal muscle undergoes a shift from local interference to coordinated systemic catabolism. Rather than accumulating solely as a maladaptive byproduct within muscle, adipose tissue depletion and remodelling actively participate in a hypercatabolic programme that accelerates muscle wasting. Longitudinal and mechanistic studies indicate that adipose tissue loss plays a critical role as one of the key interacting components in muscle atrophy. Enhanced adipocyte lipolysis, inflammatory activation and immune‐cell infiltration drive sustained lipid mobilization, increasing circulating free fatty acids and pro‐inflammatory mediators that sensitize skeletal muscle to proteolytic signalling [64, 65]. In parallel, adipose tissue remodelling—including browning‐like phenotypic changes—contributes to elevated energy expenditure and systemic energy imbalance [S23]. Consequently, in cachexia, adipose tissue no longer represents a static local impediment to regeneration but emerges as an active driver that integrates metabolic, inflammatory and endocrine cues to exacerbate muscle loss.

**FIGURE 1:**
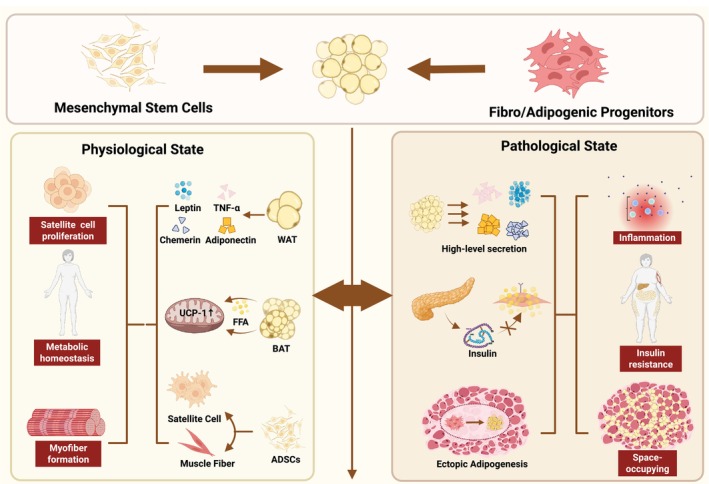
Dual roles of adipose tissue in muscle repair. Adipose depots exert context‐dependent effects on injured muscle: Beneficial actions include (i) paracrine signalling that supports myogenic responses, (ii) browning that optimizes local metabolism and (iii) adipose‐derived stem/stromal cells directly supplying progenitors for repair; detrimental actions arise from ectopic adipogenesis with fat accumulation, adipokine‐driven chronic inflammation and insulin resistance, and a space‐occupying effect that limits muscle function and regeneration. ADSCs, adipose‐derived stem/stromal cells; BAT, beige adipose tissue; WAT, white adipose tissue. Created with BioRender.com.

Collectively, these findings support a dynamic model of adipose tissue remodelling across physiological and pathological states. Under physiological conditions, adipose tissue located within and adjacent to skeletal muscle derives from tightly regulated progenitor pools and is transiently maintained, providing metabolic and paracrine support that facilitates muscle development, homeostasis and efficient regeneration. In chronic muscle disorders, such as sarcopenia and Duchenne muscular dystrophy, dysregulated progenitor fate decisions, impaired cellular clearance and persistent inflammation drive progressive expansion and functional reprogramming of adipose tissue within skeletal muscle. This maladaptive remodelling disrupts local metabolic homeostasis, amplifies inflammatory signalling and compromises the regenerative niche, ultimately promoting sustained muscle degeneration and functional decline (Figure 1; Table 1). Together, this ‘support‐versus‐inhibition’ framework highlights how intramuscular adipose tissue contextually shapes muscle repair outcomes.

**TABLE 1:** Dual roles of adipokines under physiological and pathological conditions.

	Physiological level	Pathological level
TNF‐α	TNF‐α activates p38 MAPK to promote myogenesis and satellite cell recovery acutely and limits fibrosis and fat chronically by inducing FAP apoptosis [4, 27, 34].	Persistent TNF‐α activates NF‐κB and the ubiquitin–proteasome system, degrading MyoD, blocking myogenesis, accelerating proteolysis and driving muscle wasting [59, 60].
IL‐6	Acute local IL‐6 triggers STAT3 in satellite cells, driving expansion and myogenic differentiation that add myonuclei and promote regeneration/hypertrophy [35, 36], [S30].	High‐level IL‐6 impairs muscle repair by chronically activating JAK/STAT3, which upregulates proteolysis and suppresses protein synthesis [31, 66], [S31].
Leptin	Leptin supports normal muscle mass and strength by boosting PGC‐1α and suppressing FoxO3a to drive mitochondrial biogenesis and protein synthesis, whereas FNDC5/irisin, NO and tuned STAT3 signalling promote myoblast differentiation and regeneration [28, 29, 30, 67, 68].	Excess leptin drives macrophage M1 polarization, elevating inflammatory/catabolic mediators (e.g., IL‐6) that sustain chronic inflammation and proteolysis; sustained systemic JAK/STAT and MEK activation further impairs myogenesis [14, 31, 54, 55, 69].
Adiponectin	Adiponectin, via AdipoR1–AMPK, confers anti‐inflammatory, antioxidant and metabolic benefits—boosting insulin sensitivity, mitochondrial function and fatty‐acid oxidation—and, through T‐cadherin, activates satellite cells to drive myofiber regeneration, limiting necrosis and improving remodelling [6, 32, 33, 70, 71], [S32].	Elevated adiponectin → ↓strength, ↑sarcopenia risk; AdipoRon shrinks C2C12/fast‐twitch myotubes and protein content, suggesting overactivation inhibits protein synthesis [56, 57, 58].
Chemerin	Chemerin → CMKLR1 drives myogenesis and maintains satellite‐cell function; its metabolic regulation enhances the microenvironment, indirectly supporting regeneration [42, 43].	Chemerin excess → insulin resistance and ↑CRP; in vitro, blocks myogenesis and promotes C2C12 adipogenic transdifferentiation; in metabolic syndrome, may aggravate muscle dysfunction [61, 62, 63].
Resistin	Resistin acts as a host defence peptide in innate immunity, directly participating in antimicrobial defence and the regulation of the inflammatory microenvironment [47, 48, 72].	Elevated resistin tracks with systemic inflammation and, in older adults, correlates with lower muscle mass/strength and higher sarcopenia risk. Mechanistically, it impairs myogenesis and accelerates wasting by inducing pro‐inflammatory cytokines and disrupting muscle metabolism [49, 50, 51, 52, 53, 73].

## 5 Adipose Browning Holds Potential to Enhance Muscle Repair and Counteract Fat Accumulation

BAT has been explored as an entry point to probe adipose–muscle interactions during repair because of its distinct thermogenic and metabolic properties. In humans, BAT is abundant in neonates but declines markedly with age and is largely restricted in adults to discrete cervical and supraclavicular depots, with no clear evidence for canonical intramuscular BAT under physiological conditions [S6]. Functionally, BAT differs fundamentally from white adipose tissue by virtue of its multilocular morphology, high mitochondrial content and UCP1‐dependent oxidation of fatty acids and glucose, enabling nonshivering thermogenesis and systemic metabolic regulation [S7]. Experimental studies suggest that BAT can modestly influence acute muscle injury and may confer protective effects in chronic degenerative contexts [74, 75, 76], [S24, S25]. However, direct evidence that classical brown adipose tissue exerts sustained or robust modulation of skeletal muscle regeneration remains limited, and its anatomically restricted distribution in adults constrains translational relevance. These limitations have shifted attention toward inducible thermogenic adipocytes arising from adipose browning, which retain BAT‐like functions while offering broader spatial distribution and regulatory accessibility.

Muscle injury is consistently accompanied by local adipose browning, characterized by the emergence of UCP1^+^ multilocular adipocytes [8, 75]. In experimental injury models, activation of beige‐fat programmes—through pharmacologic, genetic or cell‐based approaches—has been repeatedly shown to enhance muscle regeneration, limit atrophy, improve functional recovery and restrain ectopic fat accumulation, whereas genetic ablation of UCP1 abolishes these benefits [21, 22, 75, 76, 77, 78, 79]. Together, these observations indicate that inducible adipose browning constitutes a conserved adaptive response to muscle injury and provides a functional foundation for exploring the metabolic and paracrine mechanisms by which browning adipocytes shape the regenerative niche.

Through increased mitochondrial content and UCP1‐mediated oxidation of fatty acids and glucose, brown and beige adipocytes convert ectopic adipose tissue from a passive energy burden into an active metabolic sink, thereby limiting the expansion of adipocytes, reducing lipotoxic stress and improving local insulin sensitivity. Importantly, adipose tissue is not necessarily eliminated; rather, its metabolic state is functionally reprogrammed, weakening the coupling between fat accumulation and muscle dysfunction [S24, S25]. In parallel, browning adipocytes secrete a repertoire of batokines that modulate immune responses, vascular remodelling, metabolic flux and muscle stem cell activity, thereby reshaping the regenerative niche toward a pro‐repair state. For example, NRG4, enriched in beige adipocytes, promotes macrophage M2 polarization and modulates myofibroblast activity [80]; VEGF supports microvascular reconstruction in injured tissue [39]; the lipokine 12,13‐diHOME—elevated by cold exposure or exercise—facilitates fatty‐acid uptake and oxidation in skeletal muscle [81]; and locally secreted IGF‐I directly activates satellite cells, enhancing their proliferation and differentiation [82], [S26].

In chronic wasting states such as cachexia, adipose browning may become maladaptive and exacerbate muscle loss. Acquisition of brown‐like features by white adipocytes increases mitochondrial uncoupling and energy expenditure, thereby aggravating negative energy balance and limiting substrates required for muscle repair [24, 83, 84]. In parallel, browning‐associated batokines can reinforce catabolic signalling: Chronic IL‐6 elevation favours inflammation, suppresses satellite cell–mediated regeneration and activates proteolytic pathways, whereas GDF15—a marker of mitochondrial stress—has been linked to impaired mitochondrial function and reduced exercise capacity [66, 85, 86, 87, 88]. Together, these observations indicate that in cachexia and related conditions, browning is context dependent and can actively contribute to muscle wasting rather than recovery.

Although evidence remains limited, adipose browning shows therapeutic potential in the context of muscle injury. Moderate activation of brown and beige adipocytes supports muscle regeneration by functionally reprogramming ectopic adipose tissue into a metabolically active sink and by establishing a pro‐regenerative niche through batokine‐mediated regulation of immune, vascular and stem cell responses. However, in chronic wasting states such as cachexia, excessive or dysregulated browning may exacerbate muscle atrophy by increasing energy expenditure and inflammatory signalling. Accordingly, therapeutic strategies targeting browning must be context specific and metabolically informed, recognizing browning as a bidirectional process that requires precise control.

### 5.1 Target FAP Apoptosis, Suppress Adipogenesis and Modulate Adipokines

**FIGURE 2:**
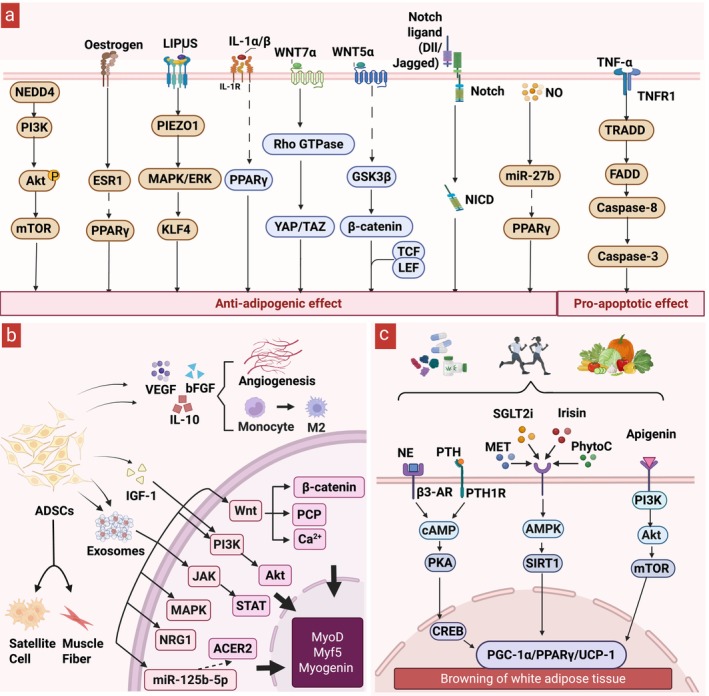
Three therapeutic strategies to promote muscle repair and attenuate fatty infiltration after muscle injury. (a) Precise regulation of FAP apoptosis and adipogenesis inhibition; (b) adipose‐derived stem cell transplantation; (c) brown adipose tissue induction. ADSCs, adipose‐derived stem/stromal cells; PhytoC, phytochemicals; SGLT2i, SGLT2 inhibitors. Created with BioRender.com.

Therapeutic strategies targeting pathological FAP activity can be organized into two phases (Figure 2a). Early after acute injury, selective depletion of excess FAPs—exemplified by nilotinib–induced TNF‐dependent apoptosis that counterbalances TGF‐β1—helps normalize cell abundance and limit maladaptive differentiation [4]. During intermediate and late phases, pathway reprogramming aims to curb adipogenesis while preserving pro‐regenerative functions. Reported approaches include restoring NOTCH sensitivity [15]; augmenting nitric‐oxide signalling via the miR‐27b–PPARγ axis [89]; leveraging IL‐1α/β–mediated repression of FAP adipogenesis [90]; activating WNT pathways [16, 91]; engaging PIEZO1–ERK/KLF4 mechanotransduction [17]; reinforcing an oestrogen–METTL3–ESR1 feedback loop [92]; maintaining thyroid hormone/D2 balance [93]; and endocrine modulation such as GIPR blockade [94]. Several agents inhibit FAP adipogenesis, including the HDAC inhibitor trichostatin A (TSA), retinoic‐acid receptor agonists, azathioprine (AKT–mTOR inhibition), metformin (ULK1‐dependent autophagy), tanshinone IIA (WNT/β‐catenin activation), glycyrrhizin and modulation of PI3K/AKT/mTOR through NEDD4 [91, 95, 96, 97, 98, 99, 100]. Together, combining early, selective clearance with later, multi‐pathway reprogramming provides a rationale for phased, individualized interventions that both reduce FAP burden and constrain adipogenic potential while preserving regenerative competence.

Once excess adipocyte accumulation is restrained—through threshold‐based control, selective FAP apoptosis and anti‐adipogenic interventions—the beneficial actions of adipokines may be more effectively revealed and harnessed. Although direct evidence remains limited, it is plausible that, under controlled ectopic adipogenesis, adipokines such as leptin, adiponectin and VEGF could operate within a defined therapeutic window to improve immune regulation, promote angiogenesis and enhance satellite cell activation, thereby supporting muscle repair. Importantly, these effects are likely threshold dependent, with moderate signalling favouring regeneration and excessive or sustained activation becoming detrimental. Further investigation into the molecular mechanisms regulating FAP fate, together with evaluation of the therapeutic feasibility of adipokine‐based approaches, may provide valuable insights into regenerative strategies that support muscle repair while minimizing adverse effects.

### 5.2 Stem Cell Transplantation

ADSCs are compelling candidates for muscle repair given their abundance, minimally invasive procurement, robust ex vivo expansion and multipotent capacity for myogenic, osteogenic and angiogenic differentiation (Figure 2b). In experimental models, ADSCs home to injured muscle via a platelet‐dependent mechanism, replenish FAPs, and support satellite cell–mediated regeneration; interrupting this trafficking impairs repair [11]. Across preclinical settings—including glucocorticoid‐induced atrophy and acute trauma—ADSC transplantation increases muscle mass, fibre cross‐sectional area and contractile force [18, 19].

ADSC‐derived exosomes are emerging as a tractable, cell‐free therapeutic. These vesicles, enriched in proteins, lipids and non‐coding RNAs, remodel the injury microenvironment by tempering inflammation, promoting angiogenesis, activating myogenic programmes, limiting fibrosis and reducing apoptosis. In experimental models, ADSC exosomes increase endothelial tube formation and vascular density, upregulate myogenic transcripts and promote satellite‐cell differentiation, thereby improving myofiber composition and contractile performance after injury [19], [S27, S28]. Their low immunogenicity and amenability to bioengineering further support their use as delivery vehicles for cell‐free therapy. In parallel, the soluble ADSC secretome—including VEGF, bFGF, IGF‐1 and IL‐10—enhances perfusion, constrains scarring, suppresses M1 polarization while favouring M2 macrophage states and directly supports MuSC survival and myogenic differentiation, collectively facilitating regeneration [18, 40, 101, 102].

ADSCs can also contribute to skeletal muscle repair by adopting myogenic fates. In vitro, exposure to 5‐azacytidine or co‐culture with myoblasts induces MyoD, MyoG and Desmin expression and supports formation of contractile myotubes [103, 104]. In vivo, transplanted ADSCs fuse with host myofibers and partially transdifferentiate into Pax7/MyoD double‐positive satellite cell–like cells that participate in long‐term regeneration [S29].

ADSCs also limit ectopic adipogenesis within injured muscle. In rotator cuff repair models, combining ADSC transplantation with surgical repair reduces fatty infiltration; administration of ADSC‐derived exosomes likewise decreases postoperative fatty infiltration (14.0 ± 2.9% vs. 21.8 ± 3.1% in controls) [105].

The therapeutic boundaries of ADSCs warrant careful consideration. Studies indicate that, under obesogenic or nutrient‐excess conditions, subcutaneous adipose tissue can release ADSCs into the circulation. These cells may home to skeletal muscle and adopt adipogenic fates, thereby worsening intramuscular fat accumulation. Mechanistically, downregulation of the CXCR4/CXCL12 retention axis in subcutaneous fat appears to facilitate this ‘release–migration–engraftment’ cascade [20]. Accordingly, in individuals with metabolic disease (e.g., obesity), transplantation of exogenous ADSCs may carry a risk of exacerbating fatty infiltration and should be deployed cautiously rather than indiscriminately.

### 5.3 Browning Intervention

Inducing adipose browning is a plausible strategy to enhance muscle repair (Figure 2c). β‐adrenergic receptor agonists (e.g., amibegron) activate cAMP–PKA signalling, upregulate UCP1 in FAPs and white adipocytes and shift cells toward a beige phenotype; in rotator cuff tear models this improves functional recovery and reduces fatty infiltration, with attenuated responses in aged animals [21, 46, 77]. Parathyroid hormone and PTH‐related peptide similarly promote browning and increase muscle mass and fibre cross‐sectional area in chronic rotator cuff injury models [22]. Additional pharmacologic routes include metformin, which augments lipid metabolism and restores browning via AMPK activation, and SGLT2 inhibitors (e.g., dapagliflozin), which stimulate mitochondrial biogenesis and browning through AMPK‐dependent or FGFR1–LKB1–AMPK pathways [106, 107]. Certain traditional Chinese medicine formulations (e.g., Zexie decoction) have also been reported to induce browning via GLP‐1R/cAMP/PKA/CREB signalling [108]. Collectively, these findings highlight the prospects for clinical application of pharmacologic interventions that reprogramme adipose metabolism to promote muscle regeneration and functional recovery.

Exercise, a non‐pharmacological intervention, promotes adipose browning through multiple pathways and can improve post‐injury repair quality. In murine rotator cuff tear and delayed‐repair models, high‐intensity interval training reduces fatty infiltration, increases myofiber cross‐sectional area and improves contractile function in a β‐adrenergic receptor–dependent manner [109]. Endurance training can promote browning indirectly via hypothalamic neuropeptide FF circuits and improve systemic metabolic indices, whereas resistance training primarily induces browning through upregulation of PGC‐1α and PPARγ [110]. The myokine irisin—generated by cleavage of FNDC5—has been implicated as a mediator that activates AMPK signalling in adipose tissue, upregulates UCP1 and drives browning [111]. Collectively, these data support exercise as a clinically relevant component of rehabilitation after muscle injury.

Specific dietary components and nutritional patterns also promote adipose tissue browning. Polyphenols and phytochemicals (e.g., capsaicin, sesamin, apigenin, 6‐gingerol, cardamonin) stimulate thermogenesis and mitochondrial biogenesis via TRPV1/β‐AR, β3‐AR/PKA, mTOR and AMPK/SIRT1/PGC‐1α pathways [112, 113, 114, 115, 116]. Medium‐chain triglycerides and short‐chain fatty acids can enhance sympathetic activity and mitochondrial function, increasing UCP1 and energy expenditure [117, 118]. At the dietary pattern level, intermittent fasting and ketogenic diets promote browning through gut microbiota–derived SCFAs or elevated β‐hydroxybutyrate [119, 120]. Collectively, these findings suggest that dietary modification—whether through targeted nutrients or structured dietary regimens—offers a promising and practical strategy to activate browning and thereby facilitate muscle repair.

Although recent studies have increasingly explored the role of adipose tissue browning in skeletal muscle repair, most evidence to date remains confined to animal models and in vitro experiments. Key issues—including the spatiotemporal dependence of browning mechanisms, their variability under pathological conditions and the safety of clinical translation—require further clarification. Notably, browning may exert dual effects under distinct metabolic states such as obesity, aging or cachexia, underscoring the need for precise regulation [24, 84]. Future investigations should therefore focus on delineating how browning interfaces with muscle regeneration and lipid accumulation in order to better define both its therapeutic potential and its limitations in promoting muscle repair and mitigating fatty infiltration.

## 6 Conclusion

Adipose tissue emerges as a context‐dependent determinant of skeletal muscle repair. Under physiological conditions, the presence of adipose tissue, through tightly regulated adipogenesis, provides metabolic support and paracrine cues that facilitate satellite cell activation and tissue remodelling. In contrast, chronic injury, aging and metabolic disease drive excessive adipose expansion within skeletal muscle and secretory reprogramming, disrupting the regenerative niche through mechanical interference, inflammatory amplification, metabolic dysfunction and impaired myogenesis. These observations support a threshold‐based model in which adipose tissue shifts from a supportive to an inhibitory role once functional boundaries are exceeded.

Building on this framework, therapeutic strategies discussed in this review aim to restore adipose balance within the muscle regenerative microenvironment. Targeted modulation of FAPs, controlled use of ADSCs and their exosomes, and context‐specific induction of adipose browning each offer distinct avenues to restrain pathological adipogenesis while preserving or reinstating pro‐regenerative functions.

In conclusion, adipose tissue should not be viewed solely as an obstacle to regeneration but as a plastic, spatiotemporally regulated component of the muscle regenerative niche. Defining adipose thresholds, directing FAP fate and achieving controlled adipokine and metabolic reprogramming—together with emerging strategies such as stem cell therapy, exosome engineering and inducible browning—provide a conceptual and translational framework for precision interventions. Future work integrating spatial, temporal and metabolic dimensions of adipose–muscle crosstalk will be essential for translating these insights into effective therapies that enhance muscle repair and functional recovery.

## Funding

This work was supported by the National Nature Science Foundation of China (82472585, 81901976, 82372544), Guangdong Provincial Natural Science Foundation (2023A1515011741), Guangzhou Science and Technology Program (2023A04J2348), Nanfang Hospital's Outstanding Young Talent Cultivation Program (K50906009, K50304020), and Nanfang Hospital’s Scientific Research Development Fund (K51701101).

## Conflicts of Interest

The authors declare no conflicts of interest.

## Supporting information


**Data S1:** Supporting information.
