# Supplementary material for: Dual Roles of Adipose Tissue in Skeletal Muscle Regeneration: Pro‐Regenerative Versus Maladaptive
Source: J Cachexia Sarcopenia Muscle. 2026 Apr 17;17(2):e70269. doi: 10.1002/jcsm.70269 (PMC13088154; doi:10.1002/jcsm.70269)
Supplement: Supplementary file 1 — Data S1: Supporting information. [file JCSM-17-e70269-s001.docx]

S1.Yin H, Price F, Rudnicki MA. Satellite cells and the muscle stem cell niche. Physiological reviews. 2013;93(1):23-67.

S2. Tidball JG. Mechanisms of muscle injury, repair, and regeneration. Comprehensive Physiology. 2011;1(4):2029-62.

S3. Kharraz Y, Guerra J, Mann CJ, Serrano AL, Muñoz-Cánoves P. Macrophage plasticity and the role of inflammation in skeletal muscle repair. Mediators of inflammation. 2013;2013:491497.

S4. Sambasivan R, Yao R, Kissenpfennig A, Van Wittenberghe L, Paldi A, Gayraud-Morel B, et al. Pax7-expressing satellite cells are indispensable for adult skeletal muscle regeneration. Development (Cambridge, England). 2011;138(17):3647-56.

S5. Wosczyna MN, Konishi CT, Perez Carbajal EE, Wang TT, Walsh RA, Gan Q, et al. Mesenchymal Stromal Cells Are Required for Regeneration and Homeostatic Maintenance of Skeletal Muscle. Cell reports. 2019;27(7):2029-35.e5.

S6. Cypess AM, Lehman S, Williams G, Tal I, Rodman D, Goldfine AB, et al. Identification and importance of brown adipose tissue in adult humans. The New England journal of medicine. 2009;360(15):1509-17.

S7. Cannon B, Nedergaard J. Brown adipose tissue: function and physiological significance. Physiological reviews. 2004;84(1):277-359.

S8. Marinkovic M, Fuoco C, Sacco F, Cerquone Perpetuini A, Giuliani G, Micarelli E, et al. Fibro-adipogenic progenitors of dystrophic mice are insensitive to NOTCH regulation of adipogenesis. Life science alliance. 2019;2(3).

S9. Reggio A, Spada F, Rosina M, Massacci G, Zuccotti A, Fuoco C, et al. The immunosuppressant drug azathioprine restrains adipogenesis of muscle Fibro/Adipogenic Progenitors from dystrophic mice by affecting AKT signaling. Scientific reports. 2019;9(1):4360.

S10. Reggio A, Rosina M, Krahmer N, Palma A, Petrilli LL, Maiolatesi G, et al. Metabolic reprogramming of fibro/adipogenic progenitors facilitates muscle regeneration. Life science alliance. 2020;3(3)

S11. Gorski T, Mathes S, Krützfeldt J. Uncoupling protein 1 expression in adipocytes derived from skeletal muscle fibro/adipogenic progenitors is under genetic and hormonal control. Journal of cachexia, sarcopenia and muscle. 2018;9(2):384-99.

S12. Biferali B, Bianconi V, Perez DF, Kronawitter SP, Marullo F, Maggio R, et al. Prdm16-mediated H3K9 methylation controls fibro-adipogenic progenitors identity during skeletal muscle repair. Science advances. 2021;7(23).

S13.Lanza MB, Ryan AS, Gray V, Perez WJ, Addison O. Intramuscular Fat Influences Neuromuscular Activation of the Gluteus Medius in Older Adults. Frontiers in physiology. 2020;11:614415.

S14. Rahemi H, Nigam N, Wakeling JM. The effect of intramuscular fat on skeletal muscle mechanics: implications for the elderly and obese. Journal of the Royal Society, Interface. 2015;12(109):20150365.

S15. Reiter DA, Bellissimo MP, Zhou L, Boebinger S, Wells GD, Jones DP, et al. Increased adiposity is associated with altered skeletal muscle energetics. Journal of applied physiology (Bethesda, Md : 1985). 2023;134(5):1083-92

S16. Bonetto A, Aydogdu T, Jin X, Zhang Z, Zhan R, Puzis L, et al. JAK/STAT3 pathway inhibition blocks skeletal muscle wasting downstream of IL-6 and in experimental cancer cachexia. American journal of physiology Endocrinology and metabolism. 2012;303(3):E410-21

S17. Oost LJ, Kustermann M, Armani A, Blaauw B, Romanello V. Fibroblast growth factor 21 controls mitophagy and muscle mass. Journal of cachexia, sarcopenia and muscle. 2019;10(3):630-42.

S18. Lee SJ, Lee YS, Zimmers TA, Soleimani A, Matzuk MM, Tsuchida K, et al. Regulation of muscle mass by follistatin and activins. Molecular endocrinology (Baltimore, Md). 2010;24(10):1998-2008.

S19. Gilson H, Schakman O, Kalista S, Lause P, Tsuchida K, Thissen JP. Follistatin induces muscle hypertrophy through satellite cell proliferation and inhibition of both myostatin and activin. American journal of physiology Endocrinology and metabolism. 2009;297(1):E157-64.

S20. Wu C, Zhang C, Li F, Yan Y, Wu Y, Li B, et al. Fucoxanthin Mitigates High-Fat-Induced Lipid Deposition and Insulin Resistance in Skeletal Muscle through Inhibiting PKM1 Activity. Journal of agricultural and food chemistry. 2024;72(32):18013-26.

S21. Zhang Y, Wang Y, Ming Z, Li B, Qi H, Xie H, et al. Vitamin K2 Alleviates Insulin Resistance Associated Skeletal Muscle Atrophy via the AKT/mTOR Signalling Pathway. Journal of cachexia, sarcopenia and muscle. 2025;16(3):e13840.

S22. Espino-Gonzalez E, Dalbram E, Mounier R, Gondin J, Farup J, Jessen N, et al. Impaired skeletal muscle regeneration in diabetes: From cellular and molecular mechanisms to novel treatments. Cell metabolism. 2024;36(6):1204-36.

S23. Chen X, Wu Q, Gong W, Ju S, Fan J, Gao X, et al. GRP75 triggers white adipose tissue browning to promote cancer-associated cachexia. Signal transduction and targeted therapy. 2024;9(1):253.

S24. Mouisel E, Bodon A, Noll C, Cassant-Sourdy S, Marques MA, Flores-Flores R, et al. Cold-induced thermogenesis requires neutral-lipase-mediated intracellular lipolysis in brown adipocytes. Cell metabolism. 2025;37(2):429-40.e5.

S25. Yuko OO, Saito M. Brown Fat as a Regulator of Systemic Metabolism beyond Thermogenesis. Diabetes & metabolism journal. 2021;45(6):840-52.

S26. Yamashita H, Kizaki T, Ookawara T, Sato Y, Yamamoto M, Ohira Y, et al. Is insulin-like growth factor I involved in brown adipose tissue enlargement? Life sciences. 1994;55(2):141-8.

S27. Byun SE, Sim C, Chung Y, Kim HK, Park S, Kim DK, et al. Skeletal Muscle Regeneration by the Exosomes of Adipose Tissue-Derived Mesenchymal Stem Cells. Current issues in molecular biology. 2021;43(3):1473-88.

S28. Figliolini F, Ranghino A, Grange C, Cedrino M, Tapparo M, Cavallari C, et al. Extracellular Vesicles From Adipose Stem Cells Prevent Muscle Damage and Inflammation in a Mouse Model of Hind Limb Ischemia: Role of Neuregulin-1. Arteriosclerosis, thrombosis, and vascular biology. 2020;40(1):239-54.

S29. Zhang Y, Zhu Y, Li Y, Cao J, Zhang H, Chen M, et al. Long-term engraftment of myogenic progenitors from adipose-derived stem cells and muscle regeneration in dystrophic mice. Human molecular genetics. 2015;24(21):6029-40.

S30. Serrano AL, Baeza-Raja B, Perdiguero E, Jardí M, Muñoz-Cánoves P. Interleukin-6 is an essential regulator of satellite cell-mediated skeletal muscle hypertrophy. Cell metabolism. 2008;7(1):33-44.

S31. Huang Z, Zhong L, Zhu J, Xu H, Ma W, Zhang L, et al. Inhibition of IL-6/JAK/STAT3 pathway rescues denervation-induced skeletal muscle atrophy. Annals of translational medicine. 2020;8(24):1681.

S32. Abou-Samra M, Selvais CM, Dubuisson N, Brichard SM. Adiponectin and Its Mimics on Skeletal Muscle: Insulin Sensitizers, Fat Burners, Exercise Mimickers, Muscling Pills … or Everything Together? International journal of molecular sciences. 2020;21(7).
